# Evaluating the effectiveness of a tailored multifaceted performance feedback intervention to improve the quality of care: protocol for a cluster randomized trial in intensive care

**DOI:** 10.1186/1748-5908-6-119

**Published:** 2011-10-24

**Authors:** Sabine N van der Veer, Maartje LG de Vos, Kitty J Jager, Peter HJ van der Voort, Niels Peek, Gert P Westert, Wilco C Graafmans, Nicolette F de Keizer

**Affiliations:** 1 Department of Medical Informatics, Academic Medical Center, PO Box 22660, 1100 DD Amsterdam, the Netherlands; 2 Scientific Centre for Transformation in Care and Welfare (Tranzo), University of Tilburg, PO Box 90153, 5000 LE Tilburg, the Netherlands; 3 Centre for Prevention and Health Services Research, National Institute for Public Health and the Environment, PO Box 1, 3720 BA Bilthoven, the Netherlands; 4 Onze Lieve Vrouwe Gasthuis, Department of Intensive Care, PO Box 95500, 1090 HM Amsterdam, the Netherlands; 5 IQ Scientific Institute for Quality of Healthcare, UMC St Radboud, PO Box 9101 - 114, 6500 HB Nijmegen, the Netherlands; 6 Directorate General for Health and Consumers, European Commission, B - 1049 Brussels, Belgium

## Abstract

**Background:**

Feedback is potentially effective in improving the quality of care. However, merely sending reports is no guarantee that performance data are used as input for systematic quality improvement (QI). Therefore, we developed a multifaceted intervention tailored to prospectively analyzed barriers to using indicators: the Information Feedback on Quality Indicators (InFoQI) program. This program aims to promote the use of performance indicator data as input for local systematic QI. We will conduct a study to assess the impact of the InFoQI program on patient outcome and organizational process measures of care, and to gain insight into barriers and success factors that affected the program's impact. The study will be executed in the context of intensive care. This paper presents the study's protocol.

**Methods/design:**

We will conduct a cluster randomized controlled trial with intensive care units (ICUs) in the Netherlands. We will include ICUs that submit indicator data to the Dutch National Intensive Care Evaluation (NICE) quality registry and that agree to allocate at least one intensivist and one ICU nurse for implementation of the intervention. Eligible ICUs (clusters) will be randomized to receive basic NICE registry feedback (control arm) or to participate in the InFoQI program (intervention arm). The InFoQI program consists of comprehensive feedback, establishing a local, multidisciplinary QI team, and educational outreach visits. The primary outcome measures will be length of ICU stay and the proportion of shifts with a bed occupancy rate above 80%. We will also conduct a process evaluation involving ICUs in the intervention arm to investigate their actual exposure to and experiences with the InFoQI program.

**Discussion:**

The results of this study will inform those involved in providing ICU care on the feasibility of a tailored multifaceted performance feedback intervention and its ability to accelerate systematic and local quality improvement. Although our study will be conducted within the domain of intensive care, we believe our conclusions will be generalizable to other settings that have a quality registry including an indicator set available.

**Trial registration:**

Current Controlled Trials ISRCTN50542146

## Background

To systematically monitor the quality of care and develop and evaluate successful improvement interventions, data on clinical performance are essential [1,2]. These performance data are often based on a set of quality indicators, ideally combining measures of structure, process, and outcomes of care [3,4].

Also within the domain of intensive care, several indicator sets have been developed [5-9], and numerous quality registries have been established worldwide to routinely have indicator data available on the performance of intensive care units (ICUs) [10-13]. In the Netherlands, the National Intensive Care Evaluation (NICE) quality registry was founded in 1996 by the Dutch intensive care profession with the aim to systematically and continuously monitor, assess, and compare ICU performance, and to improve the quality of ICU care based on the outcome indicators case-mix adjusted hospital mortality and length of ICU stay [13]. In 2006, this limited core data set of outcome indicators was extended to a total of eleven structure, process, and outcome indicators, adding items such as nurse-to-patient ratio, glucose regulation, duration of mechanical ventilation, and incidence of severe pressure ulcers. The extended set was developed by the Netherlands Society for Intensive Care (NVIC) in close collaboration with the NICE foundation [7].

Besides facilitating data collection and analyses, NICE-like most quality registries-also sends participants periodical feedback reports on their performance over time and in comparison with other groups of ICUs. Although feedback is potentially effective in improving the quality of care [14-16], merely sending feedback reports is no guarantee that performance data are used as input for systematic quality improvement (QI).

### Problem: barriers perceived by health care professionals to using performance feedback for systematic quality improvement

Previous systematic reviews reported potential barriers at different levels to using performance data for systematic improvement of health care, *e.g.*, insufficient data quality, no acknowledgement of the room for improvement in current practice, or lack of resources to implement quality interventions [15,16]. The results of a validated questionnaire completed by 142 health care professionals working at 54 Dutch ICUs confirmed that such barriers also exist within the context of intensive care [17]. As suggested by others [18,19], we translated these prospectively identified barriers into a multifaceted QI intervention using input from future users, expert knowledge, and evidence from literature. The table in 'Additional file 1' contains all barriers identified and how they are targeted by the intervention. We named the resulting QI program InFoQI (Information Feedback on Quality Indicators). InFoQI was developed and will be evaluated within the context of intensive care and the Dutch NICE registry. By targeting the potential barriers to using performance feedback as input for systematic QI activities at ICUs, the InFoQI program ultimately aims to improve the quality of intensive care.

### Study objectives

The study as proposed in this protocol aims to evaluate the effect of the tailored multifaceted feedback intervention on the use of performance indicator data for systematic QI at ICUs. Specific objectives include:

1. To assess the impact of the InFoQI program on patient outcome and organizational process measures of ICU care.

2. To gain insight into the barriers and success factors that affected the program's impact.

3. The InFoQI program was designed to overcome the previously identified barriers to using performance indicator data as input for local QI activities. Based on this assumption we hypothesize that ICUs participating in the InFoQI program will improve the quality of their care significantly more than ICUs receiving basic feedback from the NICE registry.

The results of this study will inform those involved in providing ICU care on the feasibility of the InFoQI program and its ability to accelerate systematic, local QI at ICUs. More in general, we believe that our results might be of interest to clinicians and organizations in any setting that use a quality registry including performance indicators to continuously monitor and improve the quality of care.

## Methods

### Study design

We will execute a cluster randomized controlled trial to compare facilities participating in the InFoQI program (intervention arm) to facilities receiving basic feedback from the NICE registry (control arm). Because the InFoQI program will be implemented at the facility rather than individual level, a cluster randomized trial is the preferred design for the evaluation of the program's effectiveness [20]. Like most trials aimed at evaluating organizational interventions, our study is pragmatic [21]. To apply to current standards, the study has been designed and will be reported in accordance with the CONSORT statement [22] and the appropriate extensions [23,24].

### Setting

The setting of our study is Dutch intensive care. In the Netherlands, virtually all 94 ICUs are mixed medical-surgical closed-format units, *i.e.*, units with the intensivist as the patient's primary attending physician. The units are a mixture of academic, teaching, and nonteaching settings in urban and nonurban hospitals. In 2005, 8.4 adult ICU beds per 100,000 population were available, and 466 patients per 100,000 population were admitted to the ICU that year [25]. Currently, a representative sample of 80 ICUs-covering 85% of all Dutch ICUs-voluntarily submit the limited core data set to the NICE registry, and 46 of them collect the complete, extended quality indicator data set.

At the NICE coordination center, dedicated data managers, software engineers, and a coordinator are responsible for routine processing, storing, checking, and reporting of the data. Also, for the duration of the study, two researchers will be available to provide the InFoQI program to ICUs in the intervention arm. The availability of these resources is essential for the feasibility of our study.

### Selection of participants

All 46 ICUs that participate in NICE and (are preparing to) submit data to the registry on the extended quality indicator set will be invited to participate in our study. They should be willing and able to allocate at least two staff members for an average of four hours per month to be involved in the study. The medical manager of the ICU must sign a consent form to formalize the organization's commitment.

All patients admitted to participating ICUs during the study period will be included in the analyses. However, when evaluating the impact on patient outcomes, we will exclude admissions based on the Acute Physiology and Chronic Health Evaluation (APACHE) IV exclusion criteria [26], as well as admissions following cardiac surgery, patients who were dead on admission, and admissions with any of the case mix variables missing.

### Control arm: basic feedback from the NICE registry

The ICUs allocated to the control arm will be treated as 'regular' NICE participants. This implies they will receive basic quarterly and annual feedback reports on the registry's core outcome indicators case-mix adjusted hospital mortality and length of ICU stay. In addition, they will be sent similar, but separate, basic quarterly and annual feedback reports containing data on the extended indicator set. Also, support by the NICE data managers is available and includes data quality audits, support with data collection, and additional data analyses on request. Furthermore, they are invited to a yearly discussion meeting where they can share experiences with other NICE participants.

### Intervention arm: the InFoQI program

ICUs assigned to the intervention arm, *i.e.*, participating in the InFoQI program, will receive the same intervention as the control arm, but extended with more frequent and more comprehensive feedback, a local, multidisciplinary QI team, and two educational outreach visits (Table 1).

**Table 1:** Elements of the InFoQI program (intervention arm)

Element	Description
Feedback	• monthly report for monitoring ICU's performance over time
reports	• comprehensive quarterly report for benchmarking ICU's performance to other
	groups of ICUs
	• sent to and discussed by QI team members
Local QI team	• multidisciplinary • responsible for formulating and executing a QI action plan • monthly monitoring and discussing of performance using feedback reports • sharing main findings with rest of ICU staff
Educational outreach visits	• on-site (1) at start of study period and (2) after six months • all QI team members are present; visits guided by principal investigators • promoting use of Plan-Do-Study-Act cycle for systematic quality improvement • formulating and evaluating QI action plan based on performance data

From the prospective barriers analysis, it appeared that many barriers concerned the basic NICE feedback reports. To target the lack of case-mix correction and lack of information to initiate QI actions, the basic quarterly report will be replaced by an extended, comprehensive quarterly report that facilitates comparison of an ICU's performance with that of other ICUs, *e.g.*, by providing the median length of ICU stay for elective surgery admissions in similar-sized ICUs as a benchmark. To increase the timeliness and intensity of reporting, we also developed a monthly report focusing on monitoring an ICUs' own performance over time to facilitate local evaluation of QI initiatives, *e.g.*, by providing Statistical Process Control (SPC) charts [27]. To decrease the level of data aggregation, both the monthly and quarterly reports contain data at the level of individual patients, *e.g.*, a list of unexpected non-survivors (*i.e.*, patients who died despite their low risk of mortality). The table in 'Additional file 2' summarizes the content of the reports.

ICUs in the intervention arm will establish a local QI team, creating a formal infrastructure at their department for systematic QI. This team must consist of at least one intensivist and one nurse; a management representative and a data manager are suggested as additional members. To target the lack of motivation to change, team members should be selected based on their affinity and experience with measuring and improving quality of care and their capability to convince their colleagues to be involved in QI activities. The team's main tasks are described in a protocol and include formulating a QI action plan, monitoring of performance using the feedback reports, and initiating and evaluating QI activities (see Table 1). We estimate the minimum time investment per team member to be four hours on average per month. This estimation takes into account all activities prescribed by the InFoQI program except for the execution of the QI plan. The actual time spent will depend on the type and number of QI actions in the plan.

Each ICU will receive two on-site educational outreach visits that are aimed at increasing trust in data quality, supporting the QI team members with interpreting their performance data, identifying opportunities for improvement, and translating them into a QI action plan. The structure of the visits will be equal for all intervention ICUs and the template for the action plan will be standardized. All visits will be facilitated by the same investigators who have a non-medical background; they have been involved in the development of the extended NVIC indicator set and have several years of experience with optimization of organizational processes at the ICU. Having non-clinicians supporting the QI team will make the intervention less intrusive, and therefore less threatening to participating units. It also increases the feasibility of the study, because clinical human resources are scarce in intensive care.

### Outcome measures

We used previously collected NICE data (regarding the year 2008) to select outcome measures from the extended quality indicator set to evaluate the effectiveness of our intervention. To decrease the probability of finding positive results by chance as a result of multiple hypothesis testing [28], we limited our primary endpoints to a combination of one patient outcome and one organizational process measure. We selected the indicators that showed the largest room for improvement, *i.e.*, the largest difference between the average of top-performing centers and the average of the remaining centers [29].

Primary outcome measures will be:

1. Length of ICU stay (ICU LOS); this will be calculated as the difference in days between the time of ICU discharge and time of ICU admission. To account for patients being discharged too early, the length of stay of the first ICU admission will be prolonged with the length of stay of subsequent ICU readmissions within the same hospital admission.

2. Proportion of shifts with a bed occupancy rate above 80%; this threshold is set by the NVIC in their national organizational guideline for ICUs [30]. We will calculate the bed occupancy rate as the maximum number of patients admitted simultaneously during an eight-hour nursing shift divided by the number of operational beds in that same shift. A bed will be defined as 'operational' when it is fitted with monitoring and ventilation equipment and scheduled nursing staff.

Secondary outcome measures will be all-cause, in-hospital mortality of ICU patients, duration of mechanical ventilation, proportion of glucose measurements outside the range of 2.2 to 8.0 mmol/L, and the proportion of shifts with a nurse-to-patient ratio below 0.5.

### Data collection

We will use the existing data collection methods as currently applied by the NICE registry [31]. Most ICUs participating in NICE combine manual entry of data using dedicated software with automated data extractions from electronic patient records available in, *e.g.*, their patient data management system. Each month, participants upload their data from the local, electronic database to the central, electronic registry database. ICUs in the intervention arm that have not submitted their data at the end of a month will be reminded by phone, and assisted if necessary. Quarterly reports are provided within ten weeks after the end of a period, and monthly reports within six weeks. The NICE registry uses a framework for data quality assurance [32], including elements like periodical on-site data quality audits and automated data range and consistency checks. For each ICU, additional data checks for completeness and accuracy will be performed before, during, and after the study period using descriptive statistics.

### Sample size calculations

The minimally required number of ICUs participating in the trial was based on analysis of the NICE registry 2008 data. First, ICUs were ranked by average ICU LOS of their patients. The anticipated improvement was defined as the difference in average ICU LOS of the 33% top ranked ICUs (1.28 days) and average ICU LOS among the remaining ICUs (2.11 days), and amounted to a reduction of 0.58 days per patient. A senior intensivist confirmed that this reduction is considered clinically relevant. Assuming an average number of 343 admissions per ICU per year, calculations based on the normal distribution showed that we will need at least 26 ICUs completing the trial to detect this difference with 80% power at a type I error risk (α) of 5%, taking an estimated intra-cluster correlation of 0.036 into account. With this number of ICUs, the study will also be sufficiently powered to detect a reduction in mechanical ventilation duration of 0.75 days per patient (from 2.96 to 1.75 days). We do not expect to be able to detect an effect of the intervention on ICU or hospital mortality.

To determine the required sample size for bed occupancy, shifts with an occupancy exceeding 80% were counted. This occurred in 44% of all shifts in 2008. Following the same ranking procedure as described above, a reduction of 24% was anticipated, and considered clinically relevant. Power calculations based on the binomial distribution showed that we will need a minimum of 16 ICUs completing the trial to detect this difference, taking an estimated intra-cluster correlation of 0.278 into account.

### Randomization

**Figure 1:**
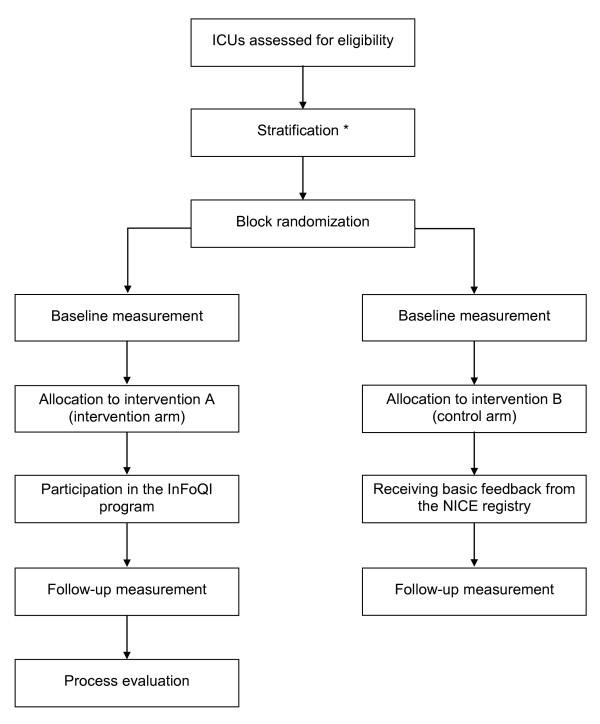
**Study flow **. * Stratification was based on size (more/less than the national median number of ventilated, non-cardiac surgery admissions) and involvement (yes/no) in a pilot to evaluate feasibility of indicator data collection.

We will randomly allocate ICUs (clusters) to one of both study arms, stratified by the number of ventilated, non-cardiac surgery admissions (less than the national median versus more than the national median), and involvement in a previous pilot study to evaluate feasibility of data collection of the NVIC indicator set [7] (involved versus not involved). Each stratum will consist of blocks with a randomly assigned size of either two or four ICUs (see Figure 1). A researcher-not involved in the study and blinded to the identity of the units-will use dedicated software to generate a randomization scheme with an equal number of interventions and controls for each block. The size and the randomization scheme of the blocks will be concealed to the investigators enrolling and assigning the ICUs. In an email to the ICU confirming the arm to which they have been allocated, the researcher that executed the randomization process will be sent this information in copy as an additional check on the assignment process. Due to the character of the intervention, it will not be possible to blind participants or the investigators providing the InFoQI program.

### Statistical analysis

For ICUs in the intervention group, the time from randomization to the first outreach visit-with an expected duration of six to eight weeks-will be regarded as a baseline period. Follow-up will end three months after the last report has been sent, assuming this is the average time required for an ICU to read, discuss, and act on a feedback report. The expected duration for intervention ICUs will therefore be approximately fourteen months. Control ICUs will have a fixed baseline period of two months, and a follow-up of fourteen months.

To assess the effect of the InFoQI program, the outcome values measured during the follow-up period will be compared between both study arms. To assess the effect of the program on length of stay, we will perform a survival analysis of time to alive ICU discharge with dying at the ICU as a competing risk [33], and adjusting for patient demographics, severity of illness during first 24 hours of admission, and admission type. To account for potential correlation of outcomes within ICUs, we will use generalized estimation equations with exchangeable correlation [34-36]. The same procedure will be used to analyze duration of mechanical ventilation. For all-cause mortality, logistic regression analysis will be used, adjusting for severity of illness at ICU admission by using the APACHE IV risk prediction model [26].

To assess the effect of the intervention on the proportion of shifts with a bed occupancy rate above 80%, shift-level occupancy data (0 for an occupancy rate below or equal to 80%, 1 for a rate above 80%) will be analyzed with logistic regression analysis. In this case, generalized estimation equations with an autoregressive correlation structure will be used to account for the longitudinal nature of shift occupancy observations. The same procedure will be followed to analyze the proportion of shifts with a nurse-to-patient ratio below 0.5.

To assess the effect on the proportion of out-of-range glucose measurements, multi-level logistic regression analysis will be performed where subsequent glucose measurements on the same patient are treated as time series data, and both patient-level and ICU-level intercept estimates are used to account for potential correlation of measurements within patients and within ICUs.

### Process evaluation

We will complement the quantitative trial results with the results from a process evaluation to gain insight into the barriers and success factors that affected the program's impact [37]. We will determine the actual exposure to the InFoQI program by asking all members of the local QI teams to record the time they have invested in the different study activities. We will also investigate the experiences of those exposed, and evaluate which of the barriers identified before the start of the program were actually solved, and if any other unknown barriers affected the program's impact; this might include barriers at the facility level as well as at the individual level. Data will be collected by sending an electronic questionnaire to all QI team members at the end of the study period. They will be asked to rate on a 5-point Likert scale to what extent they perceived certain barriers to using the InFoQI program for quality improvement at their ICU. In addition, we will invite delegates of the local QI teams for a focus group to discuss in more detail their experiences with the InFoQI program and the barriers they perceived.

### Ethics

The Institutional Review Board (IRB) of the Academic Medical Center (Amsterdam, the Netherlands) informed us that formal IRB approval and patient consent was not deemed necessary due to the focus of the InFoQI program on improving organizational processes; individual patients will not be directly involved. Additionally, in the Netherlands there is no need to obtain consent to use data from registries that do not contain patient-identifying information, as is the case in the NICE registry. The NICE foundation is officially registered according to the Dutch Personal Data Protection Act.

## Discussion

This paper describes the protocol of a cluster randomized trial to evaluate the effect of the InFoQI program on the quality of ICU care and a qualitative process evaluation to gain insight into the barriers and success factors that affected the program's impact. The program-tailored to prospectively identified barriers and facilitators-consists of comprehensive feedback reports, establishing a local, multidisciplinary QI team, and educational outreach visits. We expect that this multifaceted intervention will improve the quality of ICU care by enabling ICUs to overcome known barriers to using performance data as input for local QI activities.

### Strengths and weaknesses of the study design

In our study, we used the previously developed NVIC extended indicator set as the basis for our feedback intervention. Although the NVIC is the national organization representing the Dutch intensive care profession, some ICUs may still disagree with the relevancy of some of the indicators in the set. This would hinder the use of the feedback as input for local QI activities, potentially decreasing the effectiveness of the intervention. However, disagreement with the content of the indicator set was not identified as a barrier in our prospective barriers analysis. We will reassess this during the process evaluation.

Building on an existing indicator set also results in a clear strength of our study, because we are able to use the data collection methods as currently applied by the NICE registry. This will increase the feasibility of the InFoQI program, because eligible ICUs already routinely collect the necessary data items as a result of their participation in NICE; participation in the InFoQI program does not require additional data collection activities. Furthermore, the data quality assurance framework as applied by NICE increases the reliability of the data [31,38], and all recommended data quality control methods for QI projects [39] are being accounted for in our study. This will minimize the probability of missing and erroneous data.

Unfortunately, the design of the study will not allow us to quantitatively evaluate the relative effectiveness of the individual components of the InFoQI program. We considered a factorial design [40] for a separate evaluation of the impact of the comprehensive feedback reports and the outreach visits. However, the strong interconnectedness between the two elements made this difficult. Furthermore, the program aims to successfully overcome known barriers to using performance feedback for improving practice. During the development process of the InFoQI program, it became apparent that in order to achieve this a combination of strategies would be required. Also, previous reviews of the literature reported that multifaceted interventions seem to be more effective than single interventions [15,16,41]. Therefore, we will primarily focus on evaluating the effectiveness of the program as a whole; yet, the process evaluation will provide us with qualitative information on how and to what extent each program element might have contributed to this effectiveness.

As for the participants in our study, only ICUs that participate in the NICE registry, are capable of submitting indicator data, and agree to allocate resources to establish a local QI team will be eligible for inclusion. These criteria may lead to the selection of a non-representative sample of ICUs, because eligible facilities are less likely to be understaffed and more likely to have information technology (IT) support to facilitate routine collection of NICE data. This will not affect the internal validity of our results, because both study arms will consist of these early adopters. Moreover, the 'earliest adopters'-*i.e.*, the ICUs involved in the indicator pilot study [7]-should be equally distributed between intervention and control group as a result of our stratification method. However, the generalizability of our findings will be limited to ICUs that are motivated and equipped to systematically monitor and improve the quality of the care they deliver. Nevertheless, as the number of ICUs participating in NICE is rapidly increasing, IT in hospitals is expanding, and applying QI principles is becoming more common in health care, we believe that this requirement will not reduce the relevancy of our results for future ICU practice.

### Relation to other studies

The effectiveness of feedback as a QI strategy has often been evaluated, as indicated by the large number of included studies in systematic reviews on this subject [14,15]. However, the number of studies comparing the effect of feedback alone with the effect of feedback combined with other strategies was limited and relatively few evaluations regarded the ICU domain [14,42].

Previous before-after studies found a moderate effect of performance feedback [43] and of multidisciplinary QI teams [44] on the quality and costs of ICU care. However, many have advocated the need for rigorous evaluations using an external control group to evaluate the effect of QI initiatives [45-47], with the cluster randomized trial usually being the preferred method [48,49]. There have been cluster RCTs in the ICU domain that evaluated a multifaceted intervention with audit and feedback as a basic element [50-52]. Some of them were highly successful in increasing adherence to a specific evidence-based treatment, such as the delivery of surfactant therapy to neonates [51] and semi-recumbent positioning to prevent ventilator-associated pneumonia [50]. Our study will adopt a similar approach, combining feedback with other strategies to establish change. Nevertheless, the InFoQI program will not focus on promoting the uptake of one specific type of practice. Instead, we assume that: an ICU will be prompted to modify practice when they receive feedback on their performance being low or inconsistent with that of other ICUs; the members of the QI team are capable-with support of the facilitators-to formulate effective actions based on this feedback; and the resulting customized QI plan will contain QI activities that are considered important and feasible within the local context of the ICU. With the process evaluation, we will learn if these assumptions were correct.

### Expected meaning of the study

The results of this study will inform ICU care providers and managers on the feasibility of a tailored multifaceted performance feedback intervention and its ability to accelerate systematic, local QI activities. However, the results will also be of interest to other settings where national quality registries including performance indicators are used for continuous monitoring and improving care. Furthermore, the quantitative effect measurement together with the qualitative data from the process evaluation will contribute to the knowledge on existing barriers to using indicators for improving the quality of care and how they can be effectively overcome.

## Competing interests

The authors declare that they have no competing interests.

## Authors' contributions

GW, KJ, MDV, NDK, PVDV, SVDV, and WG had the basic idea for this study and were involved in the developing the protocol. NP planned the statistical analysis. SVDV drafted the manuscript. All authors were involved in the critical revision of the paper for intellectual content and its final approval before submission.

## Authors' information

NDK is director of the NICE registry. NDK and PVDV are members of the NICE board. PVDV is chairing the Netherlands Society of Intensive Care committee on quality indicators.

## Supplementary Material

Additional file 1 **Barriers to using performance data and how they are targeted **The prospectively identified barriers to using performance data and how they are targeted by the feedback intervention Click here for file

Additional file 2 **Content of the feedback reports **Summary of the content of the quarterly and monthly InFoQI feedback reports Click here for file
