# Supplementary material for: Evaluating the effectiveness of a tailored multifaceted performance feedback intervention to improve the quality of care: protocol for a cluster randomized trial in intensive care
Source: Implement Sci. 2011 Oct 24;6:119. doi: 10.1186/1748-5908-6-119 (PMC3217909; doi:10.1186/1748-5908-6-119)
Supplement: Additional file 1 — Barriers to using performance data and how they are targeted The prospectively identified barriers to using performance data and how they are targeted by the feedback intervention [file 1748-5908-6-119-S1.DOC]

*Additional file 1 The prospectively identified barriers to using performance data and how they are targeted by the feedback intervention*

| **Barrier identified** | **Statement (from future users or literature) to illustrate the barrier** | **How the barrier is targeted by the feedback intervention** |
| --- | --- | --- |
| Lack of knowledge on how to interpret the data | ‘Another obstacle is that people are not being taught how to handle the results, how to interpret them.’ | During educational outreach visits the facilitators support the QI team in interpreting their performance data in the reports and in formulating a QI action plan |
| Lack of information to initiate QI actions | ‘You want to improve the quality, but you don’t know where to start or where the real problems lie....The current set of [outcome] indicators doesn’t give enough information.’ | The feedback reports contain extended information on six of the indicators; During educational outreach visits the facilitators support the QI team in further exploration of data in the NICE registry |
| Lack of trust in data | ‘The data are often regarded as unreliable. If you put rubbish in, you will only get rubbish out. Trust in the data is essential.’  ‘Monitoring of quality indicators does not lead to reliable benchmark data for ICUs.’ | During educational outreach visits the facilitators discuss with the QI team completeness and correctness of the data sent to the NICE registry and -if necessary- support them in formulating actions to improve their data quality. |
| Lack of statistical power for small ICUs | ‘If your ICU is small, how reliable can your data ever get?’ | Not targeted by the intervention |
| Lack of case-mix correction | ‘…what are the characteristics of my ventilated population? That can be a cause of prolonged ventilation duration.’  ‘the ‘my patients are sicker’ syndrome.’ | Besides already available case-mix corrected hospital mortality data, data are stratified based on admission type or on APACHE IV diagnosis. During educational outreach visits the facilitators support the QI team in formulating additional case-mix related analyses on data in the NICE registry |
| Level of aggregation too high | ‘For partnership practices, the [care providers] were shown prescribing data at practice level, not at the level of the individual prescriber.’ | Besides data aggregated on ICU level, the feedback reports contain data on patient or shift level for six of the indicators. |
| Insufficient timeliness | ‘…the information might not have been presented close enough to the time of decision making.’ | As the monthly reports do not contain comparisons with other ICUs, it is possible to decrease the time between the end of a period and reporting data on this period from ten (for quarterly reports ) to six weeks (for monthly reports). |
| Lack of intensity | ‘…the [care providers] received prescriber feedback letters only once.’ | In addition to the quarterly reports, the QI team receives monthly feedback reports containing their performance data presented in a different way. |

*Additional file 1 (continued)*

| **Barrier identified** | **Statement (from future users or literature) to illustrate the barrier** | **How the barrier is targeted by the feedback intervention** |
| --- | --- | --- |
| Lack of outcome expectancy | ‘…the current rates were not considered a problem.’ | During educational outreach visits the facilitators discuss with the QI team the opportunities for improvement |
| Lack of trust in QI principles | ‘It is difficult to convince staff to use continuous quality improvement principles.’ | The facilitators discuss with the QI team members the principles of systematic QI during the educational outreach visits. |
| Lack of dissemination of information | ‘…inadequate dissemination within the hospitals.’ | Each QI team member receives the feedback reports by e-mail. During educational outreach visits and in monthly reminders they are encouraged to share their findings with the rest of the staff |
| Lack of motivation | ‘As the intervention was unsolicited, the participants had not agreed to review their practice.’ | The members of the QI team should be selected based on their affinity and experience with measuring and improving quality of care and their capability to convince staff to be involved in QI activities |
| Organizational constraints | ‘Monitoring of quality indicators does not fit into the daily routines in the hospital setting.’  ‘Patient care is the main task and [QI activities are] just an extra’  ‘You will need a change of organizational culture…That will take some time to achieve.’  ‘Most of the participating [care] facilities did not have well-developed quality improvement programs with systems to support implementing changes needed in care delivery.’ | The QI team forms the organizational basis for monitoring performance and initiating QI activities. One of their tasks is formulating a QI action plan corresponding with the opportunities for improvement within their own organization. They are also asked to discuss their performance during monthly QI team meetings, using the available reports and their QI plan as a basis. They are encouraged to report their findings during regular existing staff meetings. |
| Lack of resources | ‘Monitoring of quality indicators takes too much time.’  ‘Money is a huge obstacle. Hospitals are forced to seriously cut back their expenses in the coming few years.’ | Not targeted by the intervention |
| External barriers | ‘…there is [a lack of] public awareness now of the need to [improve the quality of care]’ | Not targeted by the intervention |
