# Supplementary material for: Evaluating the effectiveness of a tailored multifaceted performance feedback intervention to improve the quality of care: protocol for a cluster randomized trial in intensive care
Source: Implement Sci. 2011 Oct 24;6:119. doi: 10.1186/1748-5908-6-119 (PMC3217909; doi:10.1186/1748-5908-6-119)
Supplement: Additional file 2 — Content of the feedback reports Summary of the content of the quarterly and monthly InFoQI feedback reports [file 1748-5908-6-119-S2.DOC]

*Additional file 2 Summary of the content of the InFoQI feedback reports*

| **Indicator**^a^ | **Presented as** |
| --- | --- |
| **QUARTERLY INFOQI REPORT** | |
| - Patient-to-nurse ratio - Bed occupancy | Box plots displaying three months of data, with one-week periods on x-axis. Boxes based on aggregated data from ICUs with similar number of admissions are provided as benchmark. Target value as set by NVIC^b^ is made visible in plot. Separate plots for day-, evening- and night shifts and for all shifts together. |
|  | Bar charts displaying the ICU‘s mean benchmarked against means of ICUs with similar number of admissions and of same and other levels. |
| - Length of ICU stay^c^ - Mechanical ventilation duration^c^ - Glucose regulation | Text or tables with ICU’s mean or median^d^ benchmarked against mean or median of ICUs with similar number of admissions and national mean or median |
|  | Table with ICU’s own top five APACHE IV diagnoses, based on the highest value of the indicator^e^. Benchmarked against ICUs with similar number of admissions and national value. |
|  | Table with national top ten of most frequent APACHE IV admission diagnoses. For each diagnosis the value for the indicator is presented^e^. Benchmarked against ICUs with similar number of admissions and national value. |
| - Length of ICU stay^c^ - Mechanical ventilation duration^c^ | Bar charts displaying ICU’s median benchmarked against median of ICUs of same and other levels. |
|  | Tables with ICU’s percentage of outliers benchmarked against mean percentage of ICUs with similar number of admissions and national mean. |
|  | Tables with patient-specific information. No benchmarks presented. *E.g.*, admissions with an ICU length of stay longer than national 90^th^ percentile^f^ |
| - Number of unplanned extubations - Incidence of decubitus | Text or tables with ICU’s incidence of events and incidence of events relative to total number of admissions or ventilation days benchmarked against national mean, *e.g.*, the number of unplanned extubations per 100 ventilation days |
| - Availability of intensivist (on week days and in weekends) - Strategy to prevent medication errors - Measurement of patient/family satisfaction | Text or table displaying the values that ICUs submit quarterly to NICE; benchmarked against national mean, *e.g.*, the number of hours per week day that an intensivist was present at the ICU |

*Additional file B (continued)*

| **Indicator**^a^ | **Presented as** |
| --- | --- |
| **MONTHLY INFOQI REPORT** | |
| - Patient-to-nurse ratio - Bed occupancy | Run charts displaying one month of data, with days of the month on x-axis. Target value as set by NVIC^b^ is made visible in chart. Separate charts for day-, evening- and night shifts and for all shifts together. |
|  | Table with monthly top 10 of shifts with lowest patient-to-nurse ratio (at least below 0.5) or highest bed occupancy (at least above 80%) |
| - Length of ICU stay - Mechanical ventilation duration - Glucose regulation | Statistical Process Control (SPC) charts displaying one year of data, with two-week periods on x-axis. Any identified special cause variation^g^ is shown in an accompanying table. For length of ICU stay and mechanical ventilation duration there are separate charts for different types of admissions (*e.g.*, cardiac surgery, elective non-cardiac surgery, emergency non-cardiac surgery, non-surgical, etc). Glucose regulation is expressed in four separate charts, displaying the mean glucose value, time between two subsequent glucose measurements and the number of hypo- and hyperglycemic events. |
| - Glucose regulation - Mortality | Tables with patient-specific information, such as   - all patients that were admitted with a APACHE IV adjusted mortality risk <20%, but died - all hypoglycemic events^d^ |

1. Information on case-mix corrected hospital mortality and additional bar charts on length of ICU stay are fed back in separate, already existing quarterly reports, available to intervention ICUs as well as ICUs in the control group
2. For patient-to-nurse ratio the target value is between 0.5 to 1.0 (*i.e.*, minimum of one and maximum of two patients per nurse); For bed occupancy the target value is 80%
3. Most data on length of stay and ventilation duration are reported separately for different types of admissions (*e.g.*, cardiac surgery, elective non-cardiac surgery, emergency non-cardiac surgery, non-surgical, *et al*.).
4. Glucose regulation is expressed using mean glucose value, median time between two subsequent measurements and median duration of hypo- and hyperglycemic events (*i.e.*, one or more subsequent measurements with a value <2.2. mmol/l or >8.0 mmol/l resp.)
5. For glucose regulation both the percentage of measurements with a value <2.2. mmol/l and the percentage of measurements with a value of >8.0 mmol/l relative to the total number of glucose measurements are used as values.
6. The national 90^th^ percentile is calculated using all data of the previous year of all ICUs in the NICE registry
7. Special cause variation in SPC charts expresses a significant change in the process
